# Upregulation of Receptor Interacting Protein 1 Induced by UVB Contributes to Photodamage of the Skin Through NF‐κB Pathway In Vivo and In Vitro

**DOI:** 10.1111/jocd.70082

**Published:** 2025-03-10

**Authors:** Min Wei, Mengna Li, Yi Li, Baoxi Wang, Yan Yan, Li Li

**Affiliations:** ^1^ Department of Dermatology, Plastic Surgery Hospital Chinese Academy of Medical Sciences and Peking Union Medical College Beijing China; ^2^ Department of Dermatology Shanghai Sixth People's Hospital Affiliated to Shanghai Jiao Tong University School of Medicine Shanghai China

**Keywords:** inflammation, Nec‐1, photodamage, receptor interacting protein 1, ultraviolet B

## Abstract

**Background:**

Ultraviolet (UV) B can reach the epidermis and superficial dermis of the skin, inducing sunburn, inflammation, immunosuppression, cancer, and so on. Our former research found that receptor interacting protein (RIP) 1 could be upregulated in human dermal fibroblasts(HDFs) after UVB irradiation by using two‐dimensional gel electrophoresis and matrix‐assisted laser desorption/ionization time of flight mass spectrometry techniques. Besides, our further research found that RIP1 was involved in the UVB‐induced production of ROS and MMPs in HDFs. So far, the mechanisms of skin photodamage induced by UV mainly include DNA damage, oxidative stress, inflammation, apoptosis, and necroptosis. The NF‐κB pathway can be activated eventually in the occurrence of inflammation and thus produce inflammatory cytokines such as IL‐1, TNF‐α, IL‐6, and IL‐8. However, the mechanism by which the upregulation of RIP 1 induced by UVB contributes to photodamage is still unclear.

**Aims:**

To explore the role of RIP1 in UVB‐induced skin inflammation and the related signal pathways and molecular mechanism, thus providing possible molecular markers for the diagnosis and prevention of skin photodamage.

**Methods:**

Human dermal fibroblasts were cultured in vitro from normal human skin tissues. Besides, HaCaT cell lines and Balb/c nude mice were also the research objects. First, cellular models of UVB‐induced upregulation of RIP1 were established. Then, the expression of RIP1 and localization of RIP1 before and after UVB irradiation of the cells were studied through western blot and immunofluorescence. Then, the change in the expression of the nuclear factor (NF)‐kappaB (NF‐κB) pathway, along with RIP1 in these cells before and after UVB irradiation was detected, including NF‐κB p50/p65, p‐p65, IκB and cytokines such as IL‐1, IL‐6, IL‐8, and TNF‐α. Next, the effects of the RIP1 inhibitor Nec‐1 and RIP1 siRNA on the expression of RIP1, p‐RIP1, NF‐κB p50/p65, inflammatory cytokines, and nuclear translocation of p‐p65 in vitro cells after UVB irradiation were explored. At last, the expression of RIP1 and NF‐κB pathway‐related proteins such as p65/p50 was detected by western blot and immunohistochemistry before and after UVB radiation with or without subcutaneous injection of Nec‐1 in the Balb/c nude mice were detected.

**Result:**

We provide that RIP1 involved in the photodamage of human dermal fibroblasts, HaCaT cell lines, and the skin tissues of Balb/c nude mice induced by UVB. Upregulated RIP1 induced by UVB ultimately upregulates inflammatory cytokines, including IL‐1, IL‐6, IL‐8, and TNF‐α by triggering the expression of NF‐κB p65/p50 and activating nuclear translocation of p‐p65 in cells. RIP1 inhibitor Nec‐1 or RIP1 siRNA can inhibit the function of RIP1. We first illustrate that the upregulated RIP1 induced by UVB contributes to photodamage of the skin via the NF‐κB signaling pathway in vivo and in vitro.

**Conclusion:**

The study reveals the molecular mechanism by which upregulated RIP induced by UVB contributes to the occurrence of inflammation of the skin and also provides possible molecular markers for the diagnosis and prevention of skin photodamage.

## 1 Introduction

There are direct or indirect induction of DNA damages, production of reactive oxygen species, and activation of signaling pathways of inflammation and apoptosis involved in the molecular mechanisms of UV‐induced skin photodamage [1, 2]. The NF‐κB pathway, which has been widely documented, plays a key role and eventually is activated in the inflammatory response of photodamage of the skin induced by UV [3, 4]. When inactivated, NF‐κB exists as a cytoplasmic heterodimer composed of P65 and P50 subunits and can bind to the inhibitory protein IκB. When the IKK complex is activated by upstream signal stimulation, it can be recruited to the new ubiquitin chain formed by the receptor. At the same time, IκB can be phosphorylated at Ser32 and Ser36 by the IKK complex, and the protein can be ubiquitinated and the proteasome degraded, thus relieving the inhibition of NF‐κB by IκB [5]. Subsequently, the activated NF‐κB heterodimer translocates to the nucleus, promoting the transcription of pro‐inflammatory genes and then leading to the production of cytokines such as TNF‐α, IL‐1, IL‐6, and IL‐8, which can regulate inflammation [6, 7]. Besides, the P65 can be phosphorylated directly at position 536 by IKK, which increases the nuclear transcription activity of NF‐κB [1].

The development of society gives rise to an increase in people's awareness of improving their skin conditions. However, with the destruction of the ozone layer, UV radiation has become increasingly severe, and UV‐induced skin photodamage can not only cause sunburn and aging, but also accelerate the occurrence of skin cancers. Therefore, the treatment and prevention of skin photodamage are becoming more and more urgent. The previous results of our research group showed that RIP1 in human skin fibroblasts can be upregulated by UVB irradiation. As has been documented, RIP1 can be widely involved in inflammation, apoptosis, and necroptosis. Exploring the mechanism that RIP1 functions in UV‐induced skin photodamage will not only bring benefits to the in‐depth study of the mechanism and prevention of the UV‐induced photodamage of the skin, but also help to prevent other diseases in which RIP1 participates and as a potential target.

In summary, we conducted this experiment to explore the mechanism of upregulated RIP1 induced by UVB in the photodamage of the skin and especially its connection with NF‐κB pathways.

## 2 Methods

### 2.1 Materials and Animals

The fibroblasts used in this study were extracted from the living skin tissue of healthy adults (age 22–55) during surgery in our hospital. HaCaT lines were purchased from the Chinese Academy of Sciences Cell Bank (Shanghai, China) and were certified by STR. Balb/c nude mice were all female at the age of 5–6 weeks purchased from Beijing Weitong Lihua Company. The ethics involved in this experiment have been approved by the hospital's ethics committee.

### 2.2 Cell Culture and siRNA Transfection

The cells were cultured in a 10 cm cell culture dish. The fibroblasts were cultured with DMEM medium (SH30021.01, Hyclone) containing 10% FBS (10 091 148, Gibico, Gaithersburg, MD, USA) and penicillin (100 U mg mL‐1) and streptomycin (100 mg mL‐1) (SV30010, Hyclone). The HaCaT cell lines were cultured in MEM medium (GM3102‐500ML, Genview) containing 10% FBS (10 091 148, Gibico, Gaithersburg, MD, USA), penicillin (100 U mg mL‐1) and streptomycin (100 mg mL‐1) (SV30010, Hyclone). The cells were digested with 0.25% pancreatin containing EDTA (25200‐056, Invitrogen). Small interference RNA siRNA targeting human RIP 1 and negative control siRNA NC were designed and synthesized in Guangzhou RiboBio Co. Ltd. (China). The cells were plated in a six‐well plate at a density of 2 × 10^5^ cells/mL for transfection (Guangzhou RiboBio Co. Ltd., China), and subsequent experiments were performed 48 h after transfection.

### 2.3 UVB Irradiation and Nec‐1 Addition

A Waldmann UV 208 T (HerbertWaldmann GmbH&Co. Germany) with a peak emission wavelength of 313 nm was used for the UVB irradiation, as previously reported [2].

Before exposing the cells to UVB, the culture medium was discarded in an ultra‐clean table, washed with preheated PBS for two or three times. Then, 1 mL PBS buffer at about 37°C was added to the culture dishes. After fixing the ultraviolet equipment at a specific height and sterilizing it with alcohol, we turned on the Waldmann UV 208T and switched it to the appropriate parameter of the dose of UVB. Once the equipment stabilized, the prepared petri dish was placed directly underneath it. Immediately after UVB irradiation, PBS in the culture dish was discarded. For the experimental group, Nec‐1 (Selleck Chemicals，Houston, TX, USA), which was dissolved by Dimethyl sulfoxide (D2650, Sigma‐Aldrich, St. Louis, MO, USA) at 20 mM for preparation, was then diluted by DMEM (SH30021.01, Hyclone) to the concentration needed for research and added into the culture dish. At the same time, DMEM (Hyclone) of the equivalent volume was added into the culture dish in the control group. Then, the culture dish was moved to the cell culture box at a 37°C constant temperature for further culture. As for the in vivo experiment, the Balb/c nude mice were anesthetized with pentobarbital and secured with their backs exposed to the UV. Immediately after UVB irradiation, Nec‐1 diluented by dimethyl sulfoxide was injected under the dermis of the mice at a dose of 2.5 mg/kg. As a control, an equal volume of dimethyl sulfoxide (Sigma) was injected.

### 2.4 PCR

Total RNA from cells and tissues was extracted by Trizol (15 596 026, Invitrogen, Carlsbad, CA, USA). The tissues of mice were mixed with trizol in centrifuge tubes (JX‐LG0101, Jingxin industrial development Co. Ltd., Shanghai, China) and then ground by a low‐temperature tissue grinder (JXFSTPRP‐CL, Jingxin). Then, the Revert Aid First Strand cDNA Synthesis Kit (k1622, Thermo Fisher Scientific, America) was used to convert RNA to cDNA. PCR primers were designed using Primer 5 software, BioEdit software, and NCBI (Table 1). The primer was synthesized by Beijing BGI Corporation. β‐actin was used as a reference. The KAPA SYBR FAS T qPCR Kit (KK4610, Kapa Biosystems, Wilmington, MA, USA) was applied to amplify the cDNA. Data analysis was performed using the Light Cycler 480 system (Roche, Basel, Switzerland). After the experiment, the Ct values, amplification curves, and melting curves were recorded. The relative expression of mRNA in the qPCR results was calculated and expressed by 2^−ΔΔCt^.

**TABLE 1:** The primer sequences used in this experiment.

Primer	Base sequence(5'to3')
P65‐F	ACTGTTCCCCCTCATCTTCC
P65‐R	TGCTCCTCTCGCCTGGGATGCTGCC
RIPK1‐F	GGAGGAGGCGAAGATGAT
RIPK1‐R	CCTTTTACAGAAAGCGGAGT
p50‐F	CACCGTGTAAACCAAAGCC
p50‐R	GAACCAAGAAAGGAAGCCAA
ACTB‐F	GTGACAGCAGTCGGTTGGA
ACTB‐R	AGTGGGGTGGCTTTTAGGA
TNF‐α F	GGAGGACGAACATCCAACC
TNF‐α R	GACCCTAAGCCCCCAATT
IL‐1β F	ACCCTCTGTCATTCGCTCC
IL‐1β R	CTGCTACTTCTTGCCCCCT
IL‐6 F	AATCATCACTGGTCTTTTGG
IL‐6 R	CTCTGGCTTGTTCCTCAC
IL‐8 F	CTGCAGCTCTGTGTGAAGG
IL‐8 R	GGAAAGGTTTGGAGTATGTCTT

### 2.5 Western Blot

RIPA Lysis Buffer (P0013B，Beyotime, Biotechnology Co., LTD, Shanghai, China) with PMSF(ST506，Beyotime, Biotechnology Co., LTD，Shanghai, China) was used to lyse cells and tissues. The tissues of mice were mixed with RIPA and PMSF in centrifuge tubes (JX‐LG0101, Jingxin industrial development Co. Ltd., Shanghai, China) and then ground by a low‐temperature tissue grinder (JXFSTPRP‐CL, Jingxin). The concentration of protein was tested by BCA(P0012，Beyotime, Biotechnology Co., LTD，Shanghai, China). A total of 30–40 μg protein with 5 × loading buffer (P0015L, Beyotime, Biotechnology Co., LTD，Shanghai, China) were loaded in each lane; Mini‐PROTEAN Tetra and PowerPac Basic Power Supply (Bio‐Rad Laboratories, America) were used for electrophoresis. SDS‐PAGE Gel Kit was produced by Applygern (Beijing, China) and prestained protein ladder (Thermo Fisher Scientific) was used. Then the gels were transferred onto polyvinylidene difluoride membranes (Millipore, Billerica, MA, USA) by Trans‐Blot SD Semi‐Dry Electrophoretic Transfer Cell (Bio‐Rad, Hercules, CA, USA). Five percent milk was used for blocking. After washing with TBST, primary antibodies (Abcam, Cambridge, UK) were applied following their instructions at 4°C for a night, and then the pvdf membranes were incubated with the secondary antibodies for 1 h at room temperature. The primary antibodies include anti‐RIP (ab72139), anti‐RIP (ab106393), anti‐phospho‐RIP (Ser166) (65746S, CST), anti‐NF‐κB p65(ab16502), anti‐NF‐kB p65 (phospho S536) (ab86299), anti‐NFKB1, p105, p50 (proteintech, 15 506‐1‐Ap), anti‐IκB (proteintech,10 268‐1‐Ap), anti‐GAPDH (ab8245), anti‐beta actin(ab8226), anti‐gamma H2A.X (phospho S139) (ab11174).

### 2.6 Immunofluorescence

Cells were seeded on 12 × 12 mm glass slides and then fixed with 4% paraformaldehyde (Dingguo, Beijing) after specific experimental treatment. Transparent with 0.1% Triton X‐100 and blocked with 10% goat serum (Pulilai, Beijing). Incubation with the primary antibody phospho‐RIP (Ser166) (Cell signaling technology, D8I3A#44590) in a dark box at 4°C overnight and a fluorescent secondary antibody Alexa Fluor 594 (ab150080) the next day to detect RIP1 expression. To detect the expression of p‐p65, we used a primary antibody anti‐NF‐κB p65 (ab190205) combined with fluorescence Alexa Fluor 488 for overnight incubation. Subsequently, cells were counterstained with DAPI and mounted (ZLI‐9557, Zhongshan Jinqiao ZSGB‐BIO, Beijing) and then observed under the microscope using a Leica DFC300 FX microscope (Germany).

### 2.7 Immunohistochemistry

After the tissue was fixed in 4% paraformaldehyde for 24–48 h, embedded and sliced, routinely deparaffinized, and dehydrated, 0.3% hydrogen peroxide was added for 15 min at room temperature, and then the slices were placed in 1× sodium citrate Buffer (C1031, Solarbio Life Sciences, Beijing, China), microwaved for 3 min, quickly thawed for 10–15 min, and then naturally cooled at room temperature. Subsequently, it was blocked with 10% normal goat serum blocking solution (Pulilai, Beijing, China), and incubated with anti‐RIP1 (ab106393, 1/1000) and anti‐NF‐κB p65 (ab16502, 1/1000) overnight at 4°C, and then enzyme‐labeled goat anti‐mouse/rabbit IgG polymer (PV6000, ZSGB‐BIO, Beijing, China) was added; an appropriate amount of DAB(ZSGB‐BIO) was used, counterstained with hematoxylin, and mounted the slides with neutral gum. At last, it was observed under a microscope and images were collected.

### 2.8 Statistical Analysis

The data involved in the study are expressed as mean X ± standard deviation S. Graphpad Prism software version 8.0 (Graphpad software, San Diego, CA, USA) for Windows and SPSS version 22 (IBM Corp., Armonk, NY, USA) were used to plot and analyze the data. The data between the groups were analyzed by *t* test, and the *p* value < 0.05 was considered statistically significant.

## 3 Results

### 3.1 RIP1 Can Be Upregulated by 20 mJ/cm^2^ UVB in HaCaT Cells and 40 mJ/cm^2^ UVB in HDFs

**FIGURE 1:**
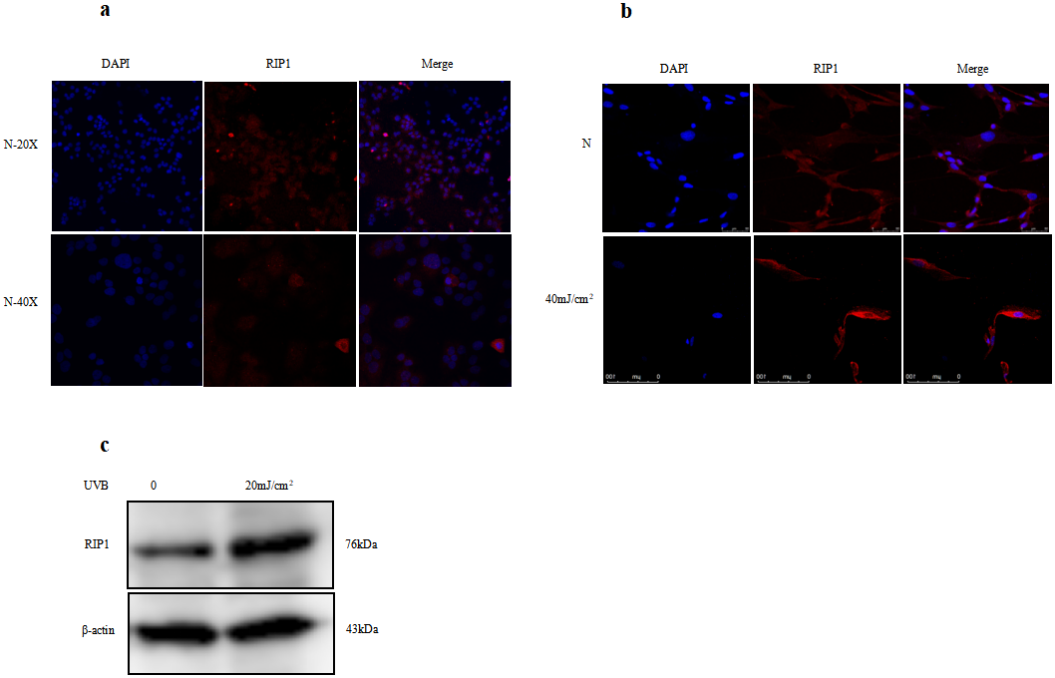
RIP1 can be upregulated by 20 mJ/cm^2^ UVB in HaCaT cells and 40 mJ/cm^2^ UVB in HDFs. (a) The expression of RIP1 in normal HaCaT cells is showed by Immunofluorescence, 20 × and 40 ×. The left panel represents the nucleus stained with 40, 6‐diamidino‐2‐phenylindole (DAPI) (blue) the middle panel represents, the localization of RIP1(red) at the plasma membrane, and the right panel represents an overlay of the middle panel and the phase contrast image. Scale bars = 100μm (*n* = 3 independent experiments). (b) The expression of RIP1 in normal HDFs and 24 h after 40 mJ/cm^2^ UVB irradiation is showed by Immunofluorescence. (c) Western blot shows that the expression of RIP1 in the HaCaT cells is significantly increased 24 h after 20 mJ/cm^2^ UVB irradiation.

Immunofluorescence was used to detect the expression of RIP1, and RIP1 was expressed in normal HaCaT and HDF cells (Figure 1a,b). RIP1 was mainly expressed in the cytoplasm of HDFs. After UVB irradiation, the expression of RIP1 shifted from the cytoplasm to the nucleus (Figure 1b).

The expression of RIP1 was significantly increased in the HaCaT cells at 24 h after being irradiated by 20 mJ/cm^2^ UVB (Figure 1c). The expression of RIP1 also significantly increased in HDF at 24 h after being irradiated by 40 mJ/cm^2^ UVB (Figure 1b). The dose of 20 mJ/cm^2^ UVB was used for the irradiation of HaCaT cells and 40 mJ/cm^2^ UVB for HDFs. The cells were collected at 24 h after irradiation for the subsequent research, so as to study the mechanism of upregulated RIP1 induced by UVB in photodamage.

### 3.2 Upregulated RIP1 by UVB May Involve Itself in Photodamage of the Skin via the NF‐κB Pathway In Vitro

**FIGURE 2:**
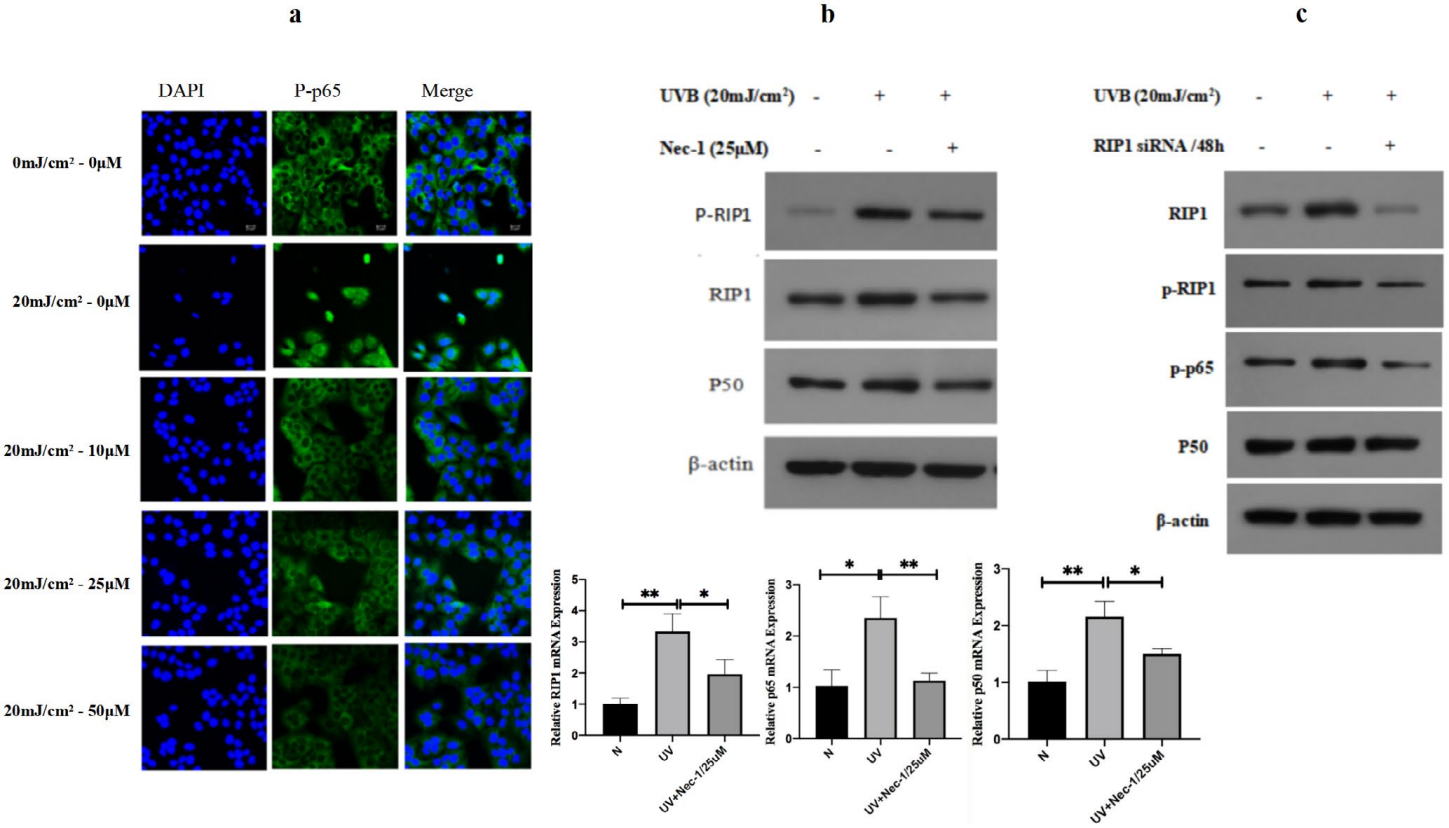
Upregulated RIP1 by UVB may involve in photodamage of the skin via NF‐κB pathway in HaCaT cells. (a) Expression localization of p‐p65 (green) without UVB irradiation or 24 h after 20 mJ/cm^2^ UVB irradiation with different concentration of Nec‐1 from 0, 10, 25, and 50 μm added in HaCaT cells is depicted. The fluorescence intensity of p‐p65 was increased in response to UVB irradiation but decreased gradually along with the increase of the concentration of Nec‐1. The magnification is 10 ×. Nuclei were stained using DAPI and indicated as blue. Scale bars = 20 μm. (b) Western blot analysis of p‐RIP1, RIP1 and NF‐κB p50 protein expression and PCR analysis of RIP1, NF‐κB p50, and p65 gene expression in HaCaT cells. (c) Protein expression of RIP1, p‐RIP1, NF‐κB p‐p65 and p50 in HaCaT cells at normal control without UVB irradiation, 24 h after UVB irradiation with or without siRNA transfected in the cells 48 h in advance UVB.

The immunofluorescence experiment was used to detect the nuclear translocation of phosphorylated p65 (p‐p65) in HaCaT cells. P‐p65 was expressed in the cytoplasm of HaCaT cells under normal conditions. While after 20 mJ/cm^2^ UVB irradiation of HaCaT cells, p‐p65 was only expressed in the nucleus (Figure 2a). In contrast, p‐p65 was expressed in the cytoplasm, and the intensity of fluorescence of p‐p65 decreased after the addition of RIP1 inhibitor (Nec‐1) in HaCaT cells, although irradiated by 20 mJ/cm^2^ UVB (Figure 2a).

Compared with the control, the protein expression of RIP1, p‐RIP1, and p65 increased significantly, while p50 increased slightly after 20 mJ/cm^2^ UVB irradiation of Nec‐1 (Figure 2b). Elevated expression of these proteins can be inhibited by the RIP1 inhibitor (Nec‐1) (Figure 2b). Besides, NF‐κB p50 and p65 gene expression in HaCaT cells also elevated after 20 mJ/cm^2^ UVB irradiation and decreased after 25 μm Nec‐1 was added, although the HaCaT cells were irradiated by 20 mJ/cm^2^ UVB (Figure 2b). The expressions of RIP1, p‐RIP1, p50, and p‐p65 proteins were significantly lower in the RIP1 siRNA transfection group than in the UVB irradiation group (Figure 2c).

**FIGURE 3:**
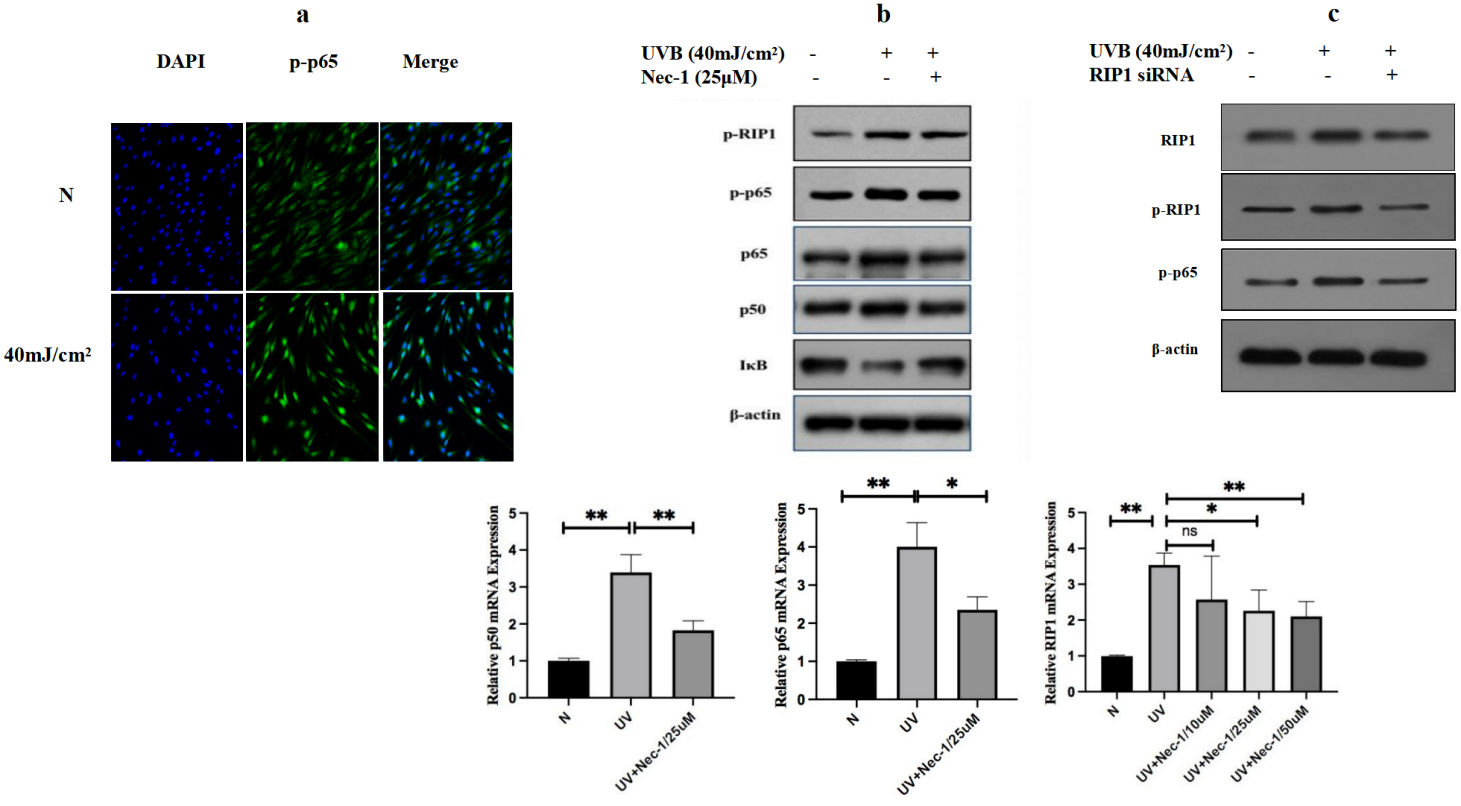
Upregulated RIP1 by UVB may involve photodamage of the skin via the NF‐κB pathway in HDFs. (a) Expression localization of p‐p65 (green) with or without UVB irradiation in HDFs is depicted. The fluorescence intensity of p‐p65 was increased in response to 40 mJ/cm^2^ UVB irradiation. Nuclei were stained using DAPI and indicated as blue. Scale bars = 50 μm. (b) Western blot and PCR analysis of the effect of Nec‐1 on the expression of RIP1 and makers of NF‐κB pathway in HDFs. (c) Protein expression of RIP1, p‐RIP1, and NF‐κB p‐p65 in HDFs at normal control without UVB irradiation, 24 h after UVB irradiation with or without siRNA transfected in the cells 48 h in advance UVB.

In similar, the HDFs were irradiated with 40 mJ/cm^2^ UVB and collected at 24 h for immunofluorescence. As is shown in Figure 3a, p‐p65 shifted from the cytoplasm to the nucleus after UVB irradiation. Besides, the gene and protein expression of RIP1 and NF‐κB p65/p50 increased in HDFs 24 h after UVB irradiation, which are markers of the inflammatory pathway (Figure 3b). The gene and protein expressions of these proteins were decreased after adding RIP1inhibitor (Nec‐1) in HDFs irradiated by 40 mJ/cm^2^ UVB (Figure 3b). Besides, Western Blot shows that RIP1 siRNA can significantly inhibit the protein expression of RIP1, p‐RIP1, and NF‐κB p‐p65 in HDFs with 40 mJ/cm^2^ UVB irradiation 48 h after RIP1 siRNA transfection (Figure 3c). Upregulated RIP1 by UVB may involve NF‐κB pathway in photodamage of skin.

### 3.3 RIP1 May Be Involved in the Production of Inflammatory Factors Induced by UVB in HaCaT Cells and HDFs

**FIGURE 4:**
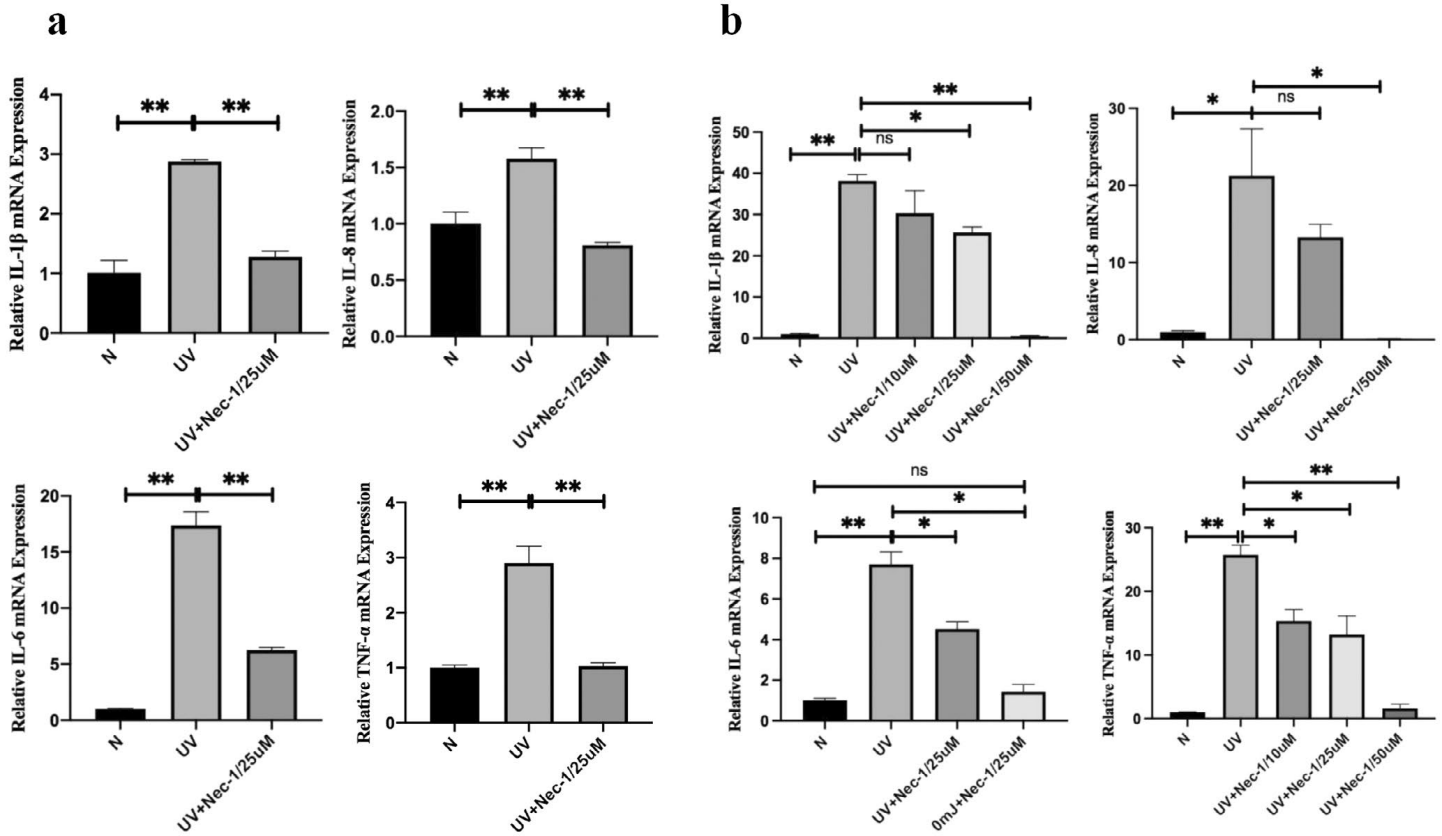
Effect of RIP1 inhibitor Nec‐1 on the gene expression of inflammatory cytokines, including IL‐1β, IL‐1, IL‐6, and TNF‐α with or without 20 mJ/cm^2^ UVB in HaCaT cells and with 25 μm Nec‐1 added in advance of UVB (a). And gene expression of IL‐1β, IL‐1, IL‐6, and TNF‐α at normal control, after 40 mJ/cm^2^ UVB in HDFs, without or with different concentrations of Nec‐1 added (b).

The production of cytokines is an important mechanism of NF‐κB involved in photodamage. RIP1 can inhibit the expression of NF‐κB induced by UVB in HaCaT cells and HDFs, which may affect the production of cytokines. The gene expression of cytokines such as IL‐1β, IL‐6, IL‐8, and TNF‐α increases significantly after exposure to UVB in HaCaT cells and HDFs. The gene expression of cytokines decreased to varying degrees after the addition of Nec‐1, and the difference was statistically significant (*p* < 0.05) (Figure 4a,b).

### 3.4 Upregulated RIP1 by UVB Involved in Photodamage of the Skin via NF‐κB Pathway In Vivo

#### 3.4.1 RIP1 and NF‐κB Can Be Upregulated in the Skin of Balb/c Mice at 48 h after being irradiated by 600 mJ/cm^2^ UVB

**FIGURE 5:**
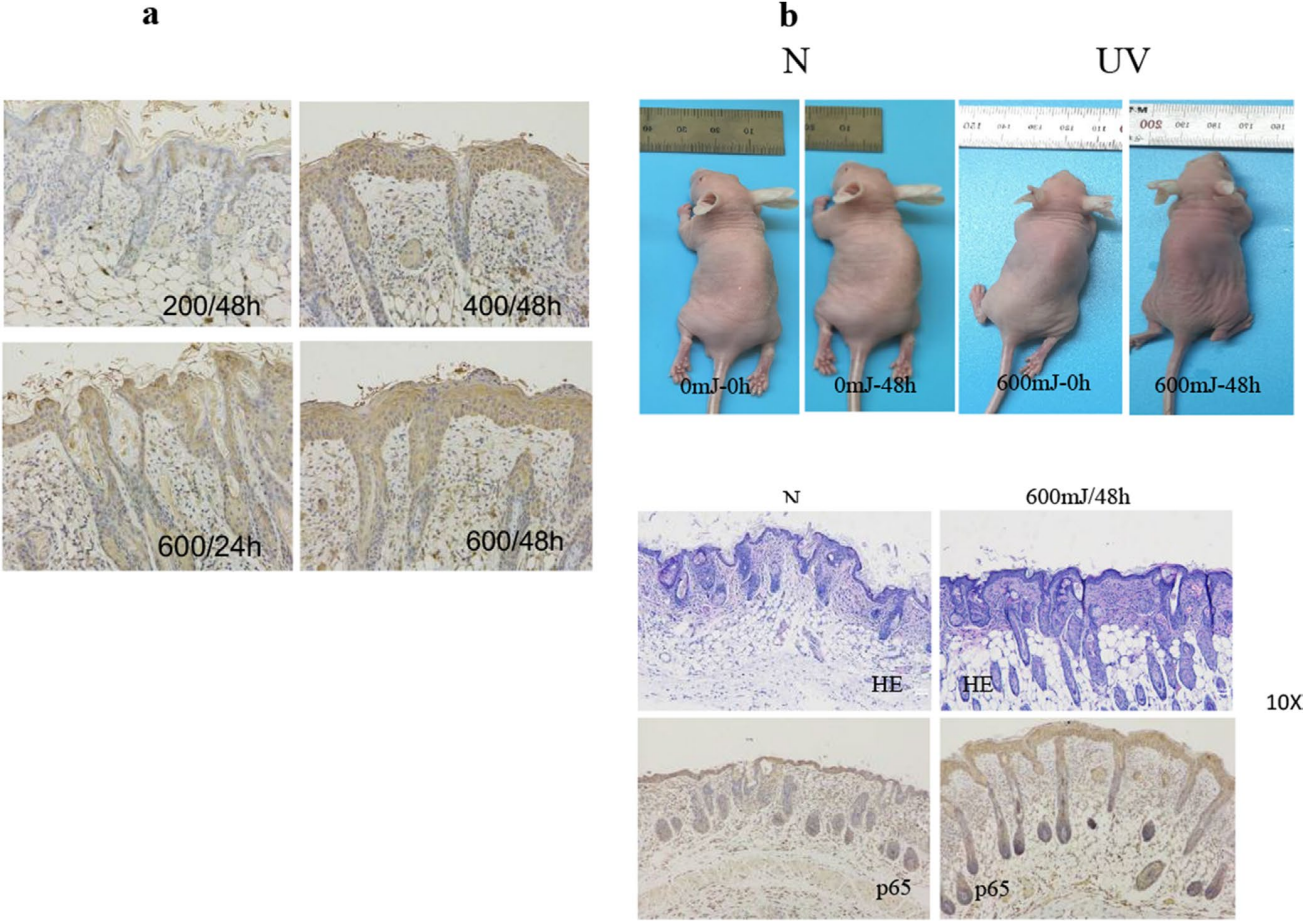
RIP1 and NF‐κB can be up‐regulated in the skin of Balb/c mice at 48 hours after irradiated by 600 mJ/cm^2^ UVB. (a) Immunohistochemical results of RIP1 protein expression of the skin tissues of Balb/c nude mice 48 h after 200, 400, and 600 mJ/cm^2^ UVB irradiation and 24 h after 600 mJ/cm^2^ irradiation. (b) Photos, HE staining, and NF‐κB p65 immunohistochemical results of Balb/c nude mice before and 48 h after 600 mJ/cm^2^ UVB irradiation.

The dorsal skin of Balb/c nude mice was irradiated by 200, 400, and 600 mJ/cm^2^ UVB. After 48 h, immunohistochemistry was employed to explore the expression of RIP1. As is shown, RIP1 is normally expressed in the skin tissue and is significantly upregulated 48 h after 600 mJ/cm^2^ UVB irradiation (Figure 5a). Then, 600 mJ/cm^2^ UVB was used to irradiate the Balb/c nude mice, and the tissues of dorsal skin were fixed for immunohistochemical analysis of the p65 expression at 48 h, which also significantly increased compared with no UVB. The photos of the mouse, HE, and immunohistochemical analysis of p65 are shown in Figure 5b.

#### 3.4.2 RIP1 Inhibitor Nec‐1 Can Inhibit UVB‐Induced Inflammation In Vivo

**FIGURE 6:**
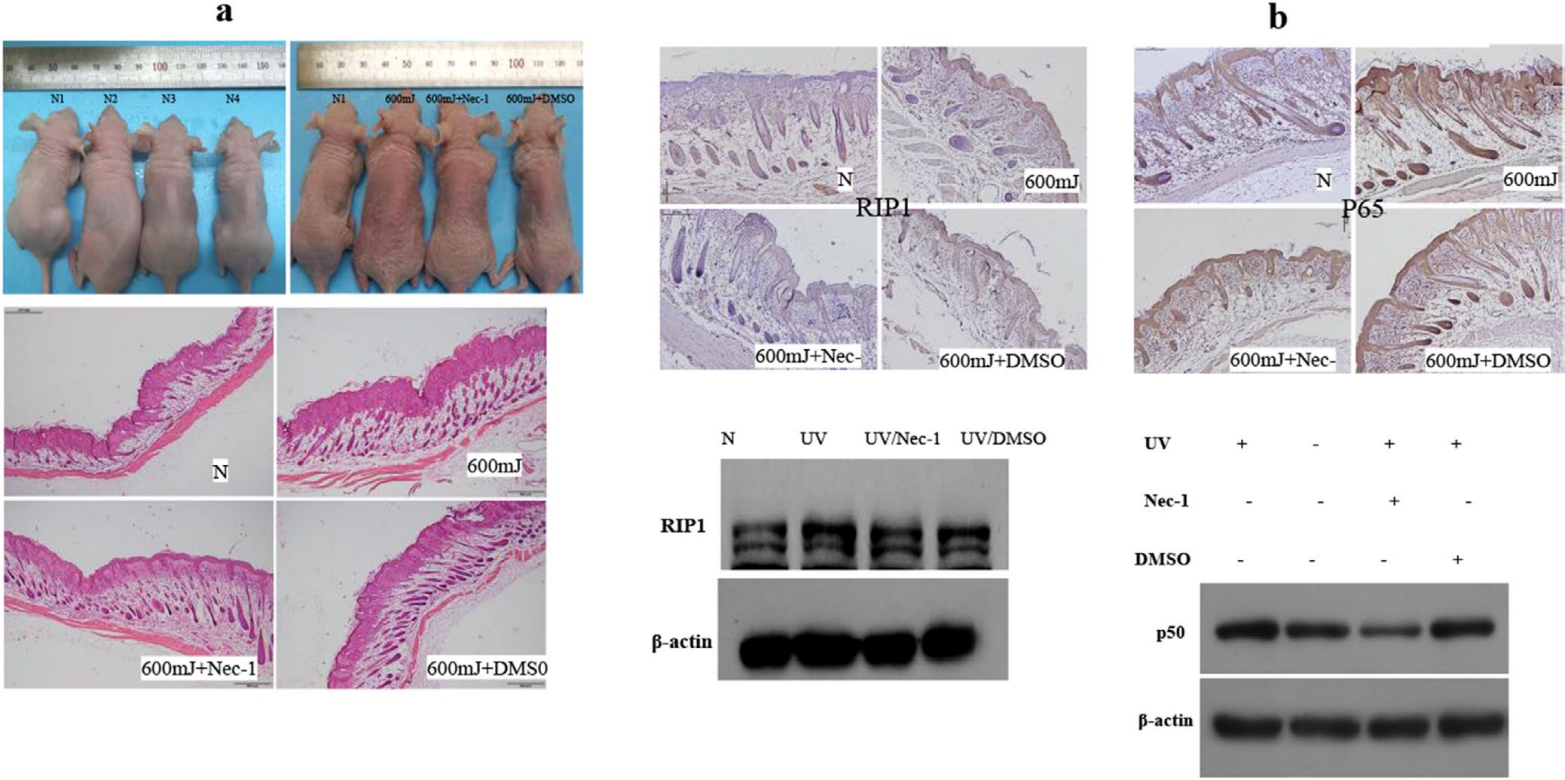
RIP1 inhibitor (Nec‐1) can inhibit UVB‐induced inflammation in vivo. (a) Photos and HE staining of Balb/c nude mice, and protein expression of RIP1 shown by immunohistochemistry and western blots of the skin tissues of the Balb/c nude mice without or with UVB, and 48 h after 600 mJ/cm^2^ UVB with subcutaneous injection of Nec‐1 or DMSO immediately after UVB irradiation. Immunohistochemistry pictures are amplified at 10 ×. Scale bar = 500 μm. (b) NF‐κB p65 immunohistochemistry and NF‐κB p50 protein expression of Balb/c nude mice skin tissue in normal condition, 48 h after 600 mJ/cm^2^ UVB with no subcutaneous injection or with Nec‐1 or DMSO injected subcutaneously immediately after UVB irradiation.

The Balb/c nude mouse were divided into four groups, including the normal control group, the UVB group exposed to 600 mJ/cm^2^, the Nec‐1 group, in which the mice were injected with Nec‐1(2.5 mg/kg) subcutaneously immediately after exposure to 600 mJ/cm^2^ UVB, and a positive control group in which DMSO was injected subcutaneously with an equal volume of the Nec‐1 after UVB. The results show that there is an obvious erythema of the skin after exposure to UVB, and erythema is slighter in the Nec‐1 group. The back skin tissues were fixed for immunohistochemistry, and the expression levels of RIP1 were also explored. It is found that the expression of RIP1 increases after exposure to UVB, and the expression of RIP1 in the Nec‐1 group significantly decreases compared to that of the normal control group. The photos of the Balb/c nude mouse, HE staining, and the immunohistochemistry and western blot results of RIP1 are shown in Figure 6a. Besides, the expression levels of p65 of the back skin tissues were also explored. The expression of NF‐κB p65 increases after exposure to UVB and decreases in the Nec‐1 group. The proteins of the dorsal skin tissues of the Balb/c nude mouse in the above four groups were extracted. The results show that, similar to RIP1, the expression of NF‐κB p50 increases 48 h after 600 mJ/cm^2^ UVB but reduces in the Nec‐1 group (Figure 6b).

## 4 Discussion

Natural light is composed of UV (about 100–380 nm), visible light (380‐780 nm) and infrared (above 780 nm) [8]. According to different wavelengths, UV can be divided into UVC(100–290 nm), UVB(290‐320 nm) and UVA(320‐400 nm) [9]. Among these, UVB and UVA can both reach the dermal layer of the skin and cause photodamage to the photodamage of the skin [10, 11]. Besides, UVB radiation is considered an important reason for sunburn and skin cancer [10]. Our group's former research found that RIP1 could be greatly upregulated after UVB irradiation in HDFs by using two‐dimensional gel electrophoresis and matrix‐assisted laser desorption/ionization time of flight mass spectrometry [12]. In another study, we found that RIP1 was involved in the production of ROS in HDFs after UVB radiation [13]. So as an active molecule in the damage to the skin, RIP1 may be a target for skin photodamage.

According to reports, RIP1 belongs to the family of receptor interacting proteins and was the “founding member” of this family, which was first discovered by Stanger BZ et al. [14]. As of now, there are seven members of the RIP family that have been discovered, which all share a homologous serine–threonine kinase domain and function as kinases and non‐kinases, and are named RIP1 to RIP7 in the order of discovery. When the external environment of the cells changes, such as during virus infection and inflammation, members of this protein family can be activated by upstream signals, triggering a series of downstream cascade‐like reactions. As has been reported, RIP1 plays a core role in inflammation, apoptosis, and necroptosis, and is widely involved in the pathogenesis of various diseases, including multiple sclerosis [15], amyotrophic lateral sclerosis [16], alzheimer's disease [17, 18], Niemann‐Pick disease [19] and hereditary retinal degeneration [20]，cancer(acute myeloid leukemia, breast cancer and colorectal cancer) and so on [21, 22]. Therefore, RIP1 is considered a potential therapeutic target for many diseases. When ubiquitinated, RIP1 can induce the activation of the downstream inflammatory signaling pathway NF‐κB. When deubiquitinated, RIP1 can bind to RIPK3 through the RHIM motif, and then together activate Caspase 8 and Caspase 3, inducing programmed apoptosis. Besides, RIP1 and RIP3 can form a necrosome, causing polyphosphorylation of the mixed lineage kinase domain‐like protein (MLKL), which can destroy the cell membrane, inducing necroptosis [23].

There are changes in fibroblasts, keratinocytes, and the redistribution of melanocytes involved in the UV‐induced photodamge of the skin [24]. In order to explore the mechanism of UVB‐induced upregulation of RIP1 in the skin, this study explored the best UVB radiation dose and time point for research and established UVB‐induced in vitro cell (HDFs, HaCaT cell lines) and animal (Balb/c nude mouse) models with upregulated RIP1 expression by using PCR, Western Blot, immunofluorescence, immunohistochemistry, and other experiments. The study results show that UVB can induce the upregulation of RIP1 expression, which is consistent with the results of our study group before. Besides, the study found that 24 h after 40 mJ/cm^2^ UVB irradiation of HDFs and 24 h after 20 mJ/cm^2^ UVB irradiation of HaCaT cell lines, 48 h after 600 mJ/cm^2^ UVB irradiation on the dorsal skin of the Balb/c nude mice can cause an increase in the expression level of RIP1 in vitro cells and Balb/c nude mouse skin. Besides, the radiation dose on the back of Balb/c nude mice is consistent with three times the minimal erythema dose reported [25].

UVB can produce its main product [(6‐4)PPs], which can trigger cell signaling pathways, activate defense systems, and lead to DNA repair or apoptosis [26]. In addition, UV radiation can upregulate mitogen‐activated protein kinase(MAPK), activate nuclear factor kappa B (NF‐κB) and activator protein 1 (AP‐1) [27, 28]. MAPK, NF‐κB and AP‐1 can inhibit collagen expression and upregulate matrix metalloproteinases (MMPs), especially MMP‐1 and ‐3, which play an important role in the degradation of extracellular matrix and cause skin wrinkles of UVB‐induced photoaging [29]. Besides, UV can also activate the mTORC2/AKT/IKKα pathway [3] and TLR/MyD88/NEMO/IKK pathway [30]，both eventually activating the NF‐κB signaling pathway.

This study also finds that UVB irradiation can cause the increase of RIP1 expression in HDFs, HaCaT cell lines, and Balb/c nude mouse skin, accompanied by the activation of the NF‐κB pathway in photodamage. With the upregulation of RIP1, the expression of NF‐kB p50/p65 mRNA and protein levels is upregulated, IκB is degraded, and p65 translocates from the cytoplasm to the nucleus. Besides, the expression of the inflammatory cytokines includes TNF‐α, IL‐1, IL‐8, IL‐6, and the photodamage markers MMP1 and MMP3 are also increased significantly. A foreign research report on inflammatory cytokines in UV‐induced damage of the mice also supports this conclusion. The report documented that 48 h after exposure to 150 mJ/cm^2^ UVB, the expression of inflammatory cytokines IL‐1β and IL‐6 of Balb/c mice skin was higher than 24 h [14]. Therefore, this study confirmed that in the acute skin damage induced by UVB, RIP1 may play an inflammatory effect through the NF‐κB pathway.

Necrostatin‐1 (Nec‐1) was discovered in 2005, when screened for small molecule compounds that can inhibit TNF‐induced necrosis in the U937 cell lines of human monocytes [31]. The molecular weight of Nec‐1 is 259.33, and the chemical formula is C_13_H_13_N_3_OS. It can occupy an allosteric lipophilic pocket of an ATP binding site in the kinase domain of RIP1 and thus hinder the binding of ATP to RIP1 [32]. As Nec‐1 can highly selectively inhibit the overexpression and autophosphorylation of RIP1 with low cytotoxicity [33], it has been widely used in the research field for RIP1‐related signaling pathways, especially in the necroptosis of various types of cells, which provides convenience for further study of the biology of RIP1 kinase [32]. Combined with other inhibitors such as RIP3 kinase inhibitor GSK‐872, Caspase8 inhibitor z‐VAD‐FMK, and MLKL inhibitor necrotic sulfonamide (NSA), Nec‐1 can finely affect and regulate the process of inflammation, apoptosis, and necroptosis.

In order to further study the regulation of RIP1 on the NF‐κB pathway, we added Nec‐1 in the cell culture medium and subcutaneously injected Nec‐1 into Babl/c nude mice to inhibit the expression and activity of RIP1 for further research. It was found that after Nec‐1 was applied, the expression of RIP1 decreased, and the expression of NF‐κB p50/p65 mRNA and protein levels and the expression of inflammatory cytokines such as IL‐1, IL‐8, and IL‐6 also decreased, too. Besides, the phenomenon of the nuclear translocation of p65 disappeared. It is proved that the RIP1 inhibitor Nec‐1 can inhibit the increase of RIP1 protein caused by UVB irradiation, thereby inhibiting the occurrence of inflammation in acute photodamage of the skin. The study can also provide a new idea for the prevention or treatment of other diseases in which RIP1 is involved. The HDFs and HaCaT cell lines transfected with RIP1 siRNA and then irradiated by UVB show that as the expression of RIP1 decreased, the activation of the NF‐κB pathway decreased, and the expression of inflammatory cytokines decreased. Once again, it is proved that the upregulated RIP1 induced by UVB in the skin can contribute to the occurrence of skin photodamage by regulating the NF‐κB signaling pathway. This discovery will not only help further research on the mechanism of photodamage and the development of targets for photoprotection, but also play an important role in the mechanism research and prevention for other light‐induced or aggravated skin diseases, such as melasma, lupus erythematosus, optical keratosis, and optical lichen planus, exogenous photodermatitis, polymorphic solar eruption, light prurigo, and so on.

This subject also has limitations. The study of the expression of RIP1 and the inflammatory reactions of the skin after UVA irradiation will also help to further understand the mechanism of the photodamage of the skin induced by UV. In addition, the specific molecular mechanism of the upregulated RIP1 induced by UVB in regulating the NF‐κB pathway still needs to be further explored.

## 5 Conclusion

UVB irradiation of HDFs, HaCaT cell lines cultured in vitro, and the skin of Balb/c nude mice can cause an increase in the protein and relative mRNA expression of RIP1. The upregulated RIP1 can activate the NF‐κB signaling pathway and thus be involved in and promote the occurrence of inflammation of skin photodamage, while the function of which can be inhibited by the RIP1 inhibitor Nec‐1 and RIP1 siRNA.

## Author Contributions

Min Wei conducted the entire experiments and prepared the original manuscript; Mengna Li helped with the experiments; Baoxi Wang and Yi Li reviewed and edited the manuscript; Yan Yan and Li Li visualized the project.

## Ethics Statement

This study was conducted in the Department of Dermatology at the Plastic Surgery Hospital, Chinese Academy of Medical Sciences and Peking Union Medical College, and was approved by the hospital institutional ethical committee.

## Conflicts of Interest

The authors declare no conflicts of interest.

## Data Availability

The data that support the findings of this study are available from the corresponding author upon reasonable request.
